# Comparative studies of the composition of bacterial microbiota associated with the ruminal content, ruminal epithelium and in the faeces of lactating dairy cows

**DOI:** 10.1111/1751-7915.12345

**Published:** 2016-02-01

**Authors:** Jun‐hua Liu, Meng‐ling Zhang, Rui‐yang Zhang, Wei‐yun Zhu, Sheng‐yong Mao

**Affiliations:** ^1^ Laboratory of Gastrointestinal MicrobiologyCollege of Animal Science and TechnologyNanjing Agricultural UniversityNanjingJiangsu ProvinceChina

## Abstract

The objective of this research was to compare the composition of bacterial microbiota associated with the ruminal content (RC), ruminal epithelium (RE) and faeces of Holstein dairy cows. The RC, RE and faecal samples were collected from six Holstein dairy cows when the animals were slaughtered. Community compositions of bacterial 16S rRNA genes from RC, RE and faeces were determined using a MiSeq sequencing platform with bacterial‐targeting universal primers 338F and 806R. UniFrac analysis revealed that the bacterial communities of RC, RE and faeces were clearly separated from each other. Statistically significant dissimilarities were observed between RC and faeces (*P* = 0.002), between RC and RE (*P* = 0.003), and between RE and faeces (*P* = 0.001). A assignment of sequences to taxa showed that the abundance of the predominant phyla Bacteroidetes was lower in RE than in RC, while a significant higher (*P* < 0.01) abundance of Proteobacteria was present in RE than in RC. When compared with the RC, the abundance of Firmicutes and Verrucomicrobia was higher in faeces, and RC contained a greater abundance of Bacteroidetes and Tenericutes. A higher proportions of *Butyrivibrio* and *Campylobacter* dominated RE as compared to RC. The faecal microbiota was less diverse than RC and dominated by genera *Turicibacter* and *Clostridium*. In general, these findings clearly demonstrated the striking compositional differences among RC, RE and faeces, indicating that bacterial communities are specific and adapted to the harbouring environment.

## Introduction

In cattle, feed digestion occurs mainly in the rumen. In the rumen, microorganisms work to convert fermented feed into volatile fatty acids and microbial mass, thus providing nutrients for the animal. Additional fermentation also occurs in the hindgut of cattle, where the fermentable substrates are limited to slower digesting polymers such as crystalline starches that have escaped foregut digestion and absorption, as well as some secreted mucins (Vanhatalo and Ketoja, 1995). Hindgut symbiotic bacteria continue the fermentation process and also provide important vitamins for the host, such as vitamin K, thiamine and riboflavin (Godoy‐Vitorino *et al*., 2012). However, feed ingredients in the hindgut are different than those in the rumen, which may result in a difference in the composition of microbial communities in the forestomach and the hindgut. Indeed, Aiple *et al*. (1992) reported that the ruminal inocula gave shorter lag times and produced more gas versus faecal inocula in *in vitro* fermentation, indicating that there are some differences in microbial activities and microbial composition between the rumen and the hindgut. In this study, we hypothesize that the hindgut may contain a different microbial community compared with the foregut; therefore, we have to compare the structure and composition of rumen and hindgut bacterial communities.

In addition to regional effects, the host's physiology may also affect the composition of gastrointestinal tract microbiota. The rumen bacterial microbiota are distinguished by three different subpopulations based on their localization: (i) the community in ruminal fluid; (ii) the community attached to solids; and (iii) the community attached to the ruminal epithelium (RE) (Cho *et al*., 2006). Among these, the ruminal epithelial adherent bacterial community performs a variety of functions necessary for host health, including the hydrolysis of urea, scavenging oxygen and recycling epithelial tissue (Cheng and Wallace, 1979). Early studies using microscopy and cultural techniques showed that bacterial community adhered to the RE were distinct from those associated with ruminal content (RC) (Cheng *et al*., 1979; McCowan *et al*., 1980). However, these culture‐based techniques may underestimate the biodiversity of the epimural biofilm, because it can be difficult to distinguish between species that are closely related, and many members of this community are likely unculturable. As a result, numerous members of the epimural community remain unidentified. The advent of genetic techniques has revealed an extensive microbial diversity that was previously undetected with culture‐dependent methods. For example, more recent work using cloning, sequencing and fingerprint profile analyses based on 16S rRNA sequences have shown both the diversity of ruminal epithelial community and its differences as compared to the community present in RC (Sadet *et al*., 2007; Sadet‐Bourgeteau *et al*., 2010; Chen *et al*., 2011). However, the information on RE is still limited because of the low throughput of the traditional 16S rRNA clone library method and fingerprint profiles. Determining 16S rRNA short variable tags using high‐throughput sequencing technologies such as 454 pyrosequencing and MiSeq sequencing provided an unprecedented sequencing depth with tens of thousands of reads per sample. These methods regenerated people's interest in measuring and comparing the composition and richness of microbial taxa in RE samples (Licht *et al*., 2007; Sayers *et al*., 2009; Mao *et al*., 2013a). Based on the 454 pyrosequencing techniques, Petri *et al*. (2013) reported that the genera *Atopobium*,* Desulfocurvus*,* Fervidicola*,* Lactobacillus* and *Olsenella* dominated the RE in beef cattle during subacute ruminal acidosis. However, the work carried out by Petri *et al*. (2013) was mainly focused on characterizing the composition of the adherent epithelial bacterial community during dietary adaptation from a forage‐based diet to a grain‐based diet, and did not explore the difference in the composition of bacterial microbiota between the RC and RE. In addition, the species composition of epithelial biofilms may be affected by other variables such as sex, diet, ruminal pH, aerotolerance, nutrient absorption, epithelial cell turnover, the passage of digesta and host communication (McCann *et al*., 2014). Moreover, the typical diet of beef cattle is different from that of dairy cattle. Thus, the composition of RE‐associated microbiota of lactating dairy cattle still needs to be explored. In this study, we characterized the composition of the microbial communities in the RE of lactating dairy cows.

## Materials and methods

### Animals and sample collection

Six Holstein dairy cattle aged 5 years (body weight: 612.9 ± 63.4 kg, milk yield: 19.4 ± 1.5 kg day^−1^, 206–287 days in lactation) were used in this study. All animal care procedures were approved by the Institutional Animal Care and Use Committee of Zhejiang University prior to initiation of the experiment. The cows' diets (at 23 kg day^−1^ dry matter intake) were formulated to meet or exceed the energy requirements of Holstein cattle yielding 25 kg of milk per day with 3.50% milk fat and 3.10% true protein (Table S1). Diets were fed *ad libitum* as a total mixed ration to reduce the selection of dietary components. The cattle were fed at 7:00 a.m. and 6:00 p.m. (one‐half of the total daily ration at each feeding). The experimental period was 86 days; the first 83 days were used for diet adaptation and the last 3 days were used for measurements. Throughout the experimental period, cattle were housed in tie stalls and fed *ad libitum*, and they were given free access to freshwater during the trial.

On day 84, 85 and 86, the cattle were slaughtered at 4–6 h after last feeding in a local slaughterhouse, with two cows slaughtered each day. The RE (50–100 g each), RC (250–300 g each) and faeces (250–300 g each) were collected within 30 min after slaughter. RE samples were rinsed three times with a sterile phosphate‐buffered saline (pH 7.0) to remove the digesta, cut into 4–5 mm^2^ samples, and scraped from the underlying tissue using a germ‐free glass slide, immediately transferred into liquid nitrogen until DNA extraction. The collected RC (250–300 g each) and faeces (250–300 g each) were homogenized in a homogenizer instrument (Media, Foshan, China) with 18000r/s for 30 s. The homogenized RC and faeces were then sampled and immediately frozen in liquid nitrogen. The remaining samples were centrifuged at 2000 *g* and the supernatants were stored at −20°C for volatile fatty acid analysis. The amount of volatile fatty acid was measured using capillary column gas chromatography (GC‐14B; Shimadzu, Kyoto, Japan; capillary column: 30 m × 0.32 mm × 0.25 mm film thickness; column temperature = 110°C; injector temperature = 180°C; detector temperature = 180°C) (Mao *et al*., 2012).

### DNA extraction, PCR amplification, illumina MiSeq sequencing and sequencing data processing

Three grams (wet weight) of homogenized samples of the RC (the ratio of rumen liquid and solid was about 1:2), RE and faeces from each cattle were used for the DNA extraction. The DNA was extracted by a bead‐beating method using a mini‐bead beater (Biospec Products, Bartlesville, OK, USA), followed by phenol–chloroform extraction (Mao *et al*., 2012). The DNA was quantified using a Nanodrop spectrophotometer (Nyxor Biotech, Paris, France) following staining using a Quant‐it Pico Green dsDNA kit (Invitrogen, Paisley, UK). The DNA samples were stored at −80°C until further processing.

The V3–V4 region of the bacteria 16S ribosomal RNA gene was amplified by PCR (95°C for 2 min, followed by 25 cycles at 95°C for 30 s, 55°C for 30 s and 72°C for 30 s with a final extension at 72°C for 5 min). The amplification used primers 338F (5′‐barcode‐ACTCCTRCGGGAGGCAGCAG‐3′) and 806R (5′‐GGACTACCVGGGTATCTAAT‐3′), where the barcode is an eight‐base sequence unique to each sample. PCR was performed in triplicate 20 μl mixtures containing 4 μl of 5× FastPfu Buffer, 2 μl of 2.5 mM dNTPs, 0.8 μl of each primer (5 μM), 0.4 μl of FastPfu Polymerase and 10 ng of template DNA. Amplicons were extracted from 2% agarose gels and purified using the AxyPrep DNA Gel Extraction Kit (Axygen Biosciences, Union City, CA, USA) according to the manufacturer's instructions and quantified using QuantiFluor^™^ ‐ST (Promega, Madison, WI, USA). Purified amplicons were pooled in equimolar concentrations and paired‐end sequenced (2 × 250) on an Illumina MiSeq platform according to standard protocols (Caporaso *et al*., 2012).

Raw FASTQ files were demultiplexed and quality‐filtered using QIIME (version 1.70) (Campbell *et al*., 2010) with the following criteria: (i) The 250 bp reads were truncated at any site receiving an average quality score < 20 over a 10 bp sliding window, and truncated reads that were shorter than 50 bp were discarded. (ii) Exact barcode matching, two nucleotide mismatch in primer matching, and reads containing ambiguous characters were removed. (iii) Only sequences that overlap longer than 10 bp were assembled according to their overlap sequence. Reads which could not be assembled were discarded. Operational taxonomic units (OTUs) were clustered with 97% similarity cut‐off using UPARSE (version 7.1 http://drive5.com/uparse/) and chimeric sequences were identified and removed using UCHIME (Edgar, 2010). The most abundant sequences within each OTU were designated as ‘representative sequences’, and were then aligned against the core set of Greengenes 13.5 (DeSantis *et al*., 2006) using PYNAST (Caporaso *et al*., 2010) with the default parameters set by QIIME. A PH Lane mask supplied by QIIME was used to remove the hypervariable regions from the aligned sequences. FASTTREE (Price *et al*., 2009) was used to create a phylogenetic tree of the representative sequences. Sequences were classified using the Ribosomal Database Project classifier with a standard minimum support threshold of 80% (Wang *et al*., 2007). Sequences identified as chloroplasts or mitochondria were removed from the analysis. Community diversity was estimated using the ACE, Chao1 and Shannon indices. The unweighted UniFrac distance method was used to perform a principal coordinate analysis (PCoA) (Lozupone and Knight, 2005), and an unweighted distance‐based analysis of molecular variance (AMOVA) was conducted to assess significant differences between the samples using mothur v.1.29.0 (Schloss *et al*., 2009).

### Use of quantitative real‐time PCR to enumerate microbial community

The 16S rRNA gene copy number of the phyla Firmicutes, and Bacteroidetes were enumerated by quantitative PCR on an Applied Biosystems 7300 Real‐Time PCR System (ABI, Foster City, CA, USA) using SYBR Green as the fluorescent dye. The reaction mixture (25 μl) consisted of 12.5 μl of IQ SYBR Green Supermix (Bio‐Rad, Richmond, CA, USA), 0.2 μM of each primer set and 5 μl of the template DNA. The amount of DNA in each sample was determined in triplicate, and the mean values were calculated. The primers were selected on the basis of a careful review of published literature. Primers for universal bacteria were forward: 5′‐CCTACGGGAGGCAGCAG‐3′ and reverse: 5′‐ATTACCGCGGCTGCTGG‐3′ (Shinkai *et al*., 2007); primers for Bacteroidetes were forward: 5′‐GGARCATGTGGTTTAATTCGATGAT‐3′ and reverse: 5′‐AGCTGACGACAACCATGCAG‐3′ (Guo *et al*., 2008); primers for Firmicutes were forward: 5′‐GGAGYATGTGGTTTAATTCGAAGCA‐3′ and reverse: 5′‐AGCTGACGACAACCATGCAC‐3′ (Zhao *et al*., 2013). External standards were prepared by making 10‐fold serial dilutions of purified plasmid DNA containing the 16S rRNA gene sequence of *Streptococcus bovis*. A standard curve was set up in every 96‐well plate and all standard curves met the required standards of efficiency (*R* ^2^ > 0.99, 90% > E > 110%). Results were expressed as log10 numbers of marker loci gene copies per gram of RC, RE or faeces (wet weight).

### Statistical analysis

The effects of sampling site on the bacterial prevalence and the VFA levels were analysed using a one‐way ANOVA procedure of SPSS (SPSS v.16; SPSS, Chicago, IL, USA) according to the following equation: Y_ij_ = μ + S_i_ + e_ij_, where Y_ij_ was the observation (VFA data, bacterial density, and the relative abundance of a given bacterial phyla, genera, or species (in %), μ was the overall mean, S_i_ was the sampling site effect (i = 3), and e_ij_ was the residual error. All *P*‐values obtained by one‐way ANOVA analyses of the bacteria community were corrected for a false discovery rate (FDR) of 0.05 with the Benjamini–Hochberg method. FDR‐corrected *P*‐values below 0.05 (*q* < 0.05) were considered significant. When *q* < 0.05, Tukey's test was employed to determine significant differences among the sampling sites.

## Results

### Diversity of the bacterial community

In this study, 16S rRNA gene sequence analysis of the RC, RE and faeces samples generated a total of 637 405 quality sequences with an average of 48 480 ± 8324 sequences per sample. The overall number of OTUs detected by the analysis was 4039 based on 97% nucleotide sequence identity between reads. To assess whether our sampling effort provided sufficient OTU coverage to accurately describe the bacterial composition of each region, sample‐based and individual‐based rarefaction curves were generated for each region (Fig. S1). The results showed that the Good's coverage was greater than 0.97, implying that our sampling effort was sufficient for the samples from all animals (Table S2). The Shannon diversity index and number of OTUs in RC were higher (*P* < 0.05) than that in RE (Table 1). The Chao value and Shannon index were also higher (*P* < 0.05) in the RC than that in the faeces. The Chao value and Shannon index were higher (*P* < 0.05) in RE than that in faeces; while the number of OTUs in RE was lower (*P* < 0.05) than that in faeces.

**Table 1:** The diversity and richness of bacterial community in the RC, RE and faeces at 3% dissimilarity level (*n* = 6)

	RC	RE	Faeces	SEM^a^	*P*‐value
Chao value	2477^a^	2439^a^	1732^b^	91	< 0.001
Shannon index	6.07^a^	5.50^b^	4.30^c^	0.18	< 0.001
Number of OTUs	1999^a^	1288^b^	1945^a^	83	< 0.001

a Standard error of means.

Means without common letter differ, *P* < 0.05.

**Figure 1:**
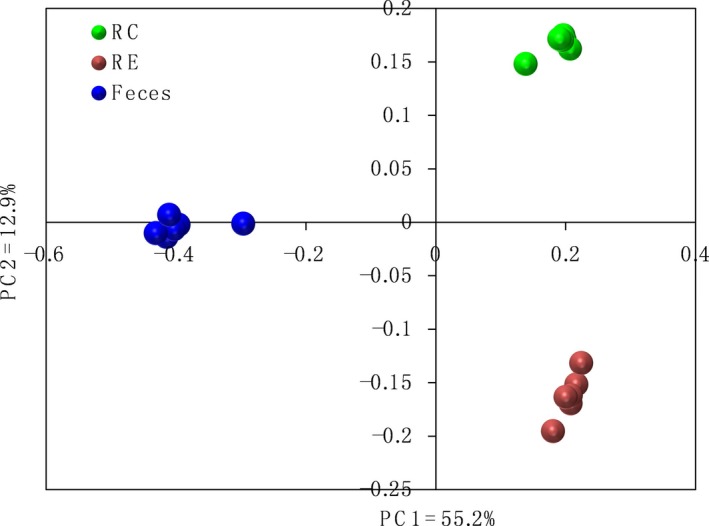
PCoA of bacterial communities from the RC, RE and faecal samples.

When the bacterial composition of microbiota among the RC, RE and faeces was compared using unweighted Unifrac distance, the bacterial communities of RC, RE and faeces clearly separated from each other (Fig. 1). AMOVA was used to assess the statistical significance of the spatial separation that was observed among the different regions in PCoA plots. Statistically significant dissimilarities were observed between RC and faeces (*P* = 0.002), between RC and RE (*P* = 0.003), and between RE and faeces (*P* = 0.001).

### The composition of the bacterial phyla, genera and OTUs

**Figure 2:**
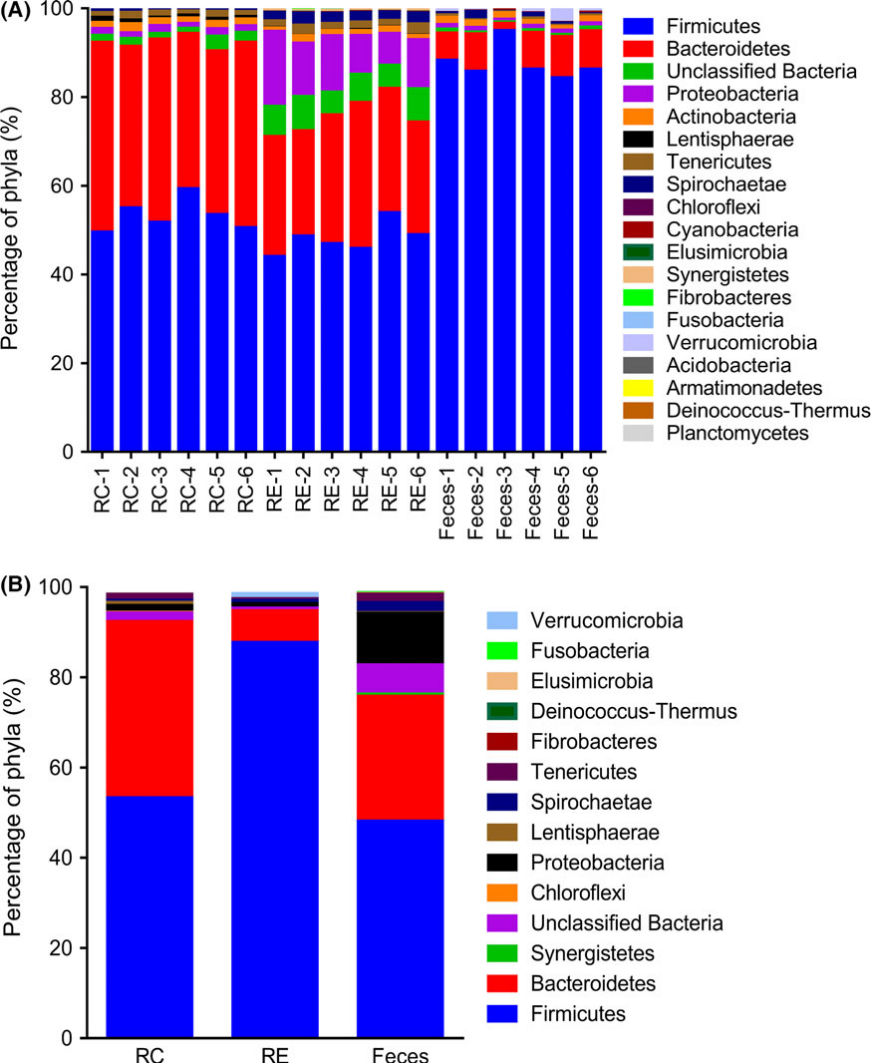
Distributions of phyla. A. The distribution of phyla for each sample. B. Distribution of the phyla averaged across the RC and RE groups (only the phyla which was significantly affected by the sampling site were presented, *q* < 0.05). C. Distribution of the phyla averaged across the RC and faeces (only the phyla which were significantly affected by the sampling site were presented, *P* < 0.05). The numbers 1 to 6 refer to the six cattle.

A total of 18 phyla were detected in all samples. Among them, Firmicutes and Bacteroidetes were detected as the dominant phyla regardless of sampling type (Fig. 2A), but their ratio among the groups varied considerably.

At the phylum level, 17 different bacterial phyla were taxonomically identified in RC samples. The majority of the sequences that were obtained belonged to Firmicutes (53.25% ± 3.55%), Bacteroidetes (39.06% ± 3.29%) and Proteobacteria (13.29% ± 0.26%) (Fig. 2A). The phyla Firmicutes, Bacteroidetes, Proteobacteria, Actinobacteria, Lentisphaerae, Tenericutes, Spirochaetae, Chloroflexi, Cyanobacteria, Elusimicrobia, Synergistetes, Fibrobacteres and Fusobacteria were found in all samples. In RE samples, 18 different bacterial phyla were identified, and the majority of the sequences that were obtained belonged to Firmicutes (48.03% ± 3.40%), Bacteroidetes (27.71% ± 3.18%) and Proteobacteria (11.43% ± 3.38%) (Fig. 2A). In faecal samples, Firmicutes (87.62% ± 3.82%) dominated all bacterial communities (Fig. 2A).

At the genus level, a total of 372 taxa were observed across all samples; however, 61.6% of all sequences were not identified at the genus level. For clarity and visualization purposes, the top 50 bacterial taxa are presented in Fig. S2. The results showed that the predominant taxa in the collected samples included *Prevotella*,* Clostridium*,* Turicibacter*,* Butyrivibrio*,* Succiniclasticum*,* Ruminococcus*,* Mogibacterium*,* Campylobacter*,* Desulfobulbus*,* Syntrophococcus*,* Acetitomaculum*,* Treponema*, unclassified Mollicutes, unclassified Ruminoco‐ccaceae, unclassified Peptostreptococcaceae, unclassified Rikenellaceae, unclassified Christensenellaceae, unclassified Prevotellaceae, unclassified Bacteroidales, unclassified Lachnospiraceae and unclassified Clostridiales.

At the OTUs level, a total of 4859 OTUs were calculated at a 0.03 dissimilarity cut‐off in combination across all of these samples. The number of OTUs shared among RC, RE and faeces was 1020 (Fig. S3). The number of OTUs shared between the RC and RE was 2444, the number of OTUs shared between the RC and faeces was 1260, whereas the number of OTUs shared between the RE and faeces was 1165. The number of OTUs specific to RC, RE and faeces was 416, 703 and 911 respectively.

### The difference in the composition of bacterial microbiota between RC and RE samples

At the phylum level, a lower (*P* < 0.05) abundance of Bacteroidetes, Lentisphaerae and Chloroflexi was observed in RE compared with RC (Fig. 2B), and the RE showed a higher (*P* < 0.05) proportion of Proteobacteria, Spirochaetae, Synergistetes, Tenericutes, Fibrobacteres, Fusobacteria, Elusimicrobia, Deinococcus‐Thermus and unclassified bacteria.

Table 2 shows the dominant taxa (the taxon with a relative abundance of ≥1% in at least one sampling site) significantly affected (*q* < 0.05) by the sampling sites. When compared with RE, the RC had a greater (*P* < 0.05) percentage of *Prevotella*,* Saccharofermentans*,* Succiniclasticum*,* Ruminococcus*, and some unclassified bacteria including unclassified Ruminococcaceae, unclassified Christensenellaceae and unclassified Bacteroidales, while RE presented a higher (*P* < 0.05) abundance of *Butyrivibrio*,* Mogibacterium*,* Treponema*,* Syntrophococcus*,* Howardella*,* Campylobacter, Desulfovibrio*,* Desulfobulbus*, unclassified Clostridiales, unclassified Prevotellaceae, unclassified bacteria and unclassified Erysipelotrichaceae.

**Table 2:** Dominant taxa calculated from collected samples of RC, RE and faeces (*n* = 6)

Classification	Percentage of total sequences			SEM^a^	*q*‐value
	RC	RE	Faeces		
Firmicutes					
*Butyrivibrio*	3.34^b^	11.32^a^	2.00^b^	1.02	< 0.001
*Clostridium*	0.01^b^	0.02^b^	12.64^a^	1.47	< 0.001
*Turicibacter*	0.02^b^	0.01^b^	7.99^a^	0.94	< 0.001
*Ruminococcus*	2.43^a^	0.35^c^	1.51^b^	0.22	< 0.001
*Cellulosilyticum*	< 0.01^b^	< 0.01^b^	1.45^a^	0.21	< 0.001
*Saccharofermentans*	1.92^a^	0.27^b^	0.07^c^	0.21	< 0.001
*Syntrophococcus*	0.18^b^	2.90^a^	0.04^b^	0.34	< 0.001
*Howardella*	0.03^b^	1.41^a^	0.02^b^	0.19	< 0.001
*Succiniclasticum*	3.65^a^	2.17^b^	< 0.01^c^	0.38	< 0.001
*Acetitomaculum*	1.92^a^	1.34^ab^	0.88^b^	0.14	0.001
*Mogibacterium*	1.22^b^	2.39^a^	0.76^c^	0.19	< 0.001
Unclassified Christensenellaceae	11.66^a^	2.41^b^	3.50^b^	1.03	< 0.001
Unclassified Ruminococcaceae	15.26^a^	7.26^b^	18.04^a^	1.37	< 0.001
Unclassified Lachnospiraceae	5.48^a^	5.81^a^	1.95^b^	0.47	< 0.001
Unclassified Clostridiales	1.99^b^	6.19^a^	1.91^b^	0.51	< 0.001
Unclassified Peptostreptococcaceae	0.13^b^	0.04^b^	29.61^a^	3.46	< 0.001
Unclassified Erysipelotrichaceae	0.32^b^	1.35^a^	0.24^b^	0.13	< 0.001
Bacteroidetes					
*Prevotella*	16.94^a^	2.29^b^	0.09^c^	1.87	< 0.001
Unclassified Rikenellaceae	10.66^a^	9.43^a^	2.26^b^	0.94	< 0.001
Unclassified Prevotellaceae	4.02^b^	10.70^a^	0.76^c^	1.05	< 0.001
Unclassified Bacteroidales	6.81^a^	4.39^b^	2.40^c^	0.51	< 0.001
Proteobacteria					
*Campylobacter*	< 0.01^b^	3.72^a^	< 0.01^b^	0.56	0.001
*Desulfovibrio*	0.04^b^	1.38^a^	< 0.01^b^	0.16	< 0.001
*Desulfobulbus*	0.01^b^	3.13^a^	< 0.01^b^	0.39	< 0.001
Spirochaetae					
*Treponema*	0.4^c^	1.95^a^	0.72^b^	0.19	< 0.001
*Verrucomicrobia*					
*Akkermansia*	ND	< 0.01^b^	1.16^a^	0.19	0.007
Tenericutes					
Unclassified Mollicutes	1.22^a^	1.57^a^	0.37^b^	0.14	< 0.001
Unclassified					
Unclassified Bacteria	1.87^b^	6.44^a^	0.58^c^	0.64	< 0.001

a Standard error of means.

Means without common letter differ, *q* < 0.05.

Of the 4859 OTUs detected in this study, 35 OTUs represented ≥ 1% of all sequences in at least one sampling site (Table 3). When compared with the RE, the relative abundance of 10 OTUs was higher in the RC (*P* < 0.05), while the RE presented a higher proportion of 16 OTUs (*P* < 0.05).

**Table 3:** Dominant OTU calculated from collected samples of RC, RE and faeces (*n* = 6)

No. OTU ID	Classification	Percentage of total sequences			SEM^a^	*q*‐value
		RC	RE	Faeces		
Bacteroidetes						
OTU1916	*Prevotella*	1.14^a^	0.18^b^	< 0.01^b^	0.13	< 0.001
OTU2537	*Prevotella*	1.54^a^	0.09^b^	ND	0.44	0.003
OTU6271	*Prevotella*	1.19^a^	0.06^b^	ND	0.82	< 0.001
OTU6764	Unclassified Prevotellaceae	0.06^b^	7.91^a^	< 0.01^c^	0.17	< 0.001
OTU3160	Unclassified Rikenellaceae	3.59^a^	0.60^b^	< 0.01^b^	0.30	< 0.001
OTU3169	Unclassified Rikenellaceae	0.02^b^	1.96^a^	ND	0.11	< 0.001
OTU5919	Unclassified Rikenellaceae	0.02^b^	1.28^a^	ND	0.16	< 0.001
Firmicutes						
OTU1531	*Butyrivibrio*	< 0.01^b^	2.47^a^	< 0.01^b^	0.56	< 0.001
OTU3217	*Butyrivibrio*	< 0.01^b^	2.48^a^	ND	0.13	< 0.001
OTU369	*Butyrivibrio*	0.85^b^	0.09^c^	1.21^a^	0.28	< 0.001
OTU6481	*Butyrivibrio*	ND	1.10	ND	0.16	< 0.001
OTU2081	*Cellulosilyticum*	< 0.01^b^	< 0.01^b^	1.11^a^	0.54	< 0.001
OTU604	*Clostridium*	0.01^b^	0.01^b^	11.60^a^	0.18	< 0.001
OTU2453	*Howardella*	0.03^b^	1.38^a^	0.02^b^	0.18	< 0.001
OTU4649	*Mogibacterium*	0.08^b^	1.71^a^	0.05^b^	0.24	< 0.001
OTU1687	*Saccharofermentans*	1.06^a^	0.15^b^	0.06^b^	0.15	< 0.001
OTU1719	*Succiniclasticum*	1.54^a^	0.43^b^	< 0.01^b^	0.40	< 0.001
OTU5108	*Succiniclasticum*	1.81^a^	1.27^b^	< 0.01^b^	0.28	< 0.001
OTU1315	*Turicibacter*	0.02^b^	0.01^b^	6.99^a^	0.29	< 0.001
OTU3497	Unclassified Christensenellaceae	1.00^a^	0.12^b^	0.05^b^	0.11	< 0.001
OTU583	Unclassified Christensenellaceae	4.70^a^	0.31^b^	0.12^b^	0.13	< 0.001
OTU1072	Unclassified Clostridiales	< 0.01^b^	1.03^a^	ND	0.12	< 0.001
OTU5630	Unclassified Clostridiales	< 0.01^b^	1.03^a^	ND	0.32	< 0.001
OTU1956	Unclassified Lachnospiraceae	0.12^b^	2.28^a^	< 0.01^b^	0.20	< 0.001
OTU2405	Unclassified Peptostreptococcaceae	0.01^b^	< 0.01^b^	1.52^a^	1.87	< 0.001
OTU4730	Unclassified Peptostreptococcaceae	0.06^b^	0.02^b^	16.00^a^	1.11	< 0.001
OTU4916	Unclassified Peptostreptococcaceae	0.06^b^	0.01^b^	9.54^a^	0.20	< 0.001
OTU120	Unclassified Ruminococcaceae	4.23^a^	1.18^b^	0.05^c^	0.12	< 0.001
OTU1888	Unclassified Ruminococcaceae	0.01^b^	< 0.01^b^	4.55^a^	0.52	< 0.001
OTU3720	Unclassified Ruminococcaceae	< 0.01^b^	< 0.01^b^	2.59^a^	0.15	< 0.001
Proteobacteria						
OTU2345	*Campylobacter*	< 0.01^b^	3.66^a^	< 0.01^b^	1.35	0.001
OTU3579	*Desulfobulbus*	< 0.01^b^	1.10^a^	ND	0.13	< 0.001
Spirochaetae						
OTU713	*Treponema*	< 0.01^b^	1.28^a^	ND	0.13	< 0.001

a Standard error of means.

Means without common letter differ, *q* < 0.05.

### The difference in the composition of bacterial microbiota between RC and faecal samples

At the phylum level, when compared with RC (Fig. 2B), the abundance of Firmicutes and Verrucomicrobia was higher (*P* < 0.05) in faeces, while the RC contained a greater abundance (*P* < 0.05) of Bacteroidetes, Chloroflexi, Lentisphaerae, Tenericutes and unclassified bacteria.

At the genus level, when compared with RC, the abundance of *Clostridium*,* Turicibacter*,* Cellulosilyticum*,* Akkermansia*,* Treponema* and unclassified Peptostreptococcaceae was greater (*P* < 0.05) in faeces (Table 2), while the percentages of *Ruminococcus*,* Acetitomaculum*,* Mogibacterium*,* Prevotella*,* Saccharofermentans*,* Succiniclasticum*, unclassified Christensenellaceae, unclassified Bacteroidales, unclassified Rikenellaceae, unclassified Lachnospiraceae, unclassified Prevotellaceae, unclassified bacteria and unclassified Mollicutes were lower (*P* < 0.05) in faeces.

At the OTUs level, when compared with faeces (Table 3), the relative abundance of 10 OTUs was higher in RC (*P* < 0.05), while faeces showed a greater abundance of nine OTUs (*P* < 0.05).

### The difference in the composition of bacterial microbiota between RE and faecal samples

At the phylum level, when compared with RE (Fig. 2B), the abundance of Firmicutes and Verrucomicrobia was higher (*P* < 0.05) in faeces, while the RE contained a greater abundance (*P* < 0.05) of Bacteroidetes, Synergistetes, unclassified bacteria, Proteobacteria, Spirochaetae, Tenericutes, Fibrobacteres, Deinococcus‐Thermus, Elusimicrobia and Fusobacteria.

At the genus level, the abundance of *Butyrivibrio*,* Campylobacter*,* Desulfobulbus*,* Desulfovibrio*,* Howardella*,* Mogibacterium*,* Prevotella*,* Succiniclasticum*,* Syntrophococcus*,* Treponema*, unclassified bacteria, unclassified Bacteroidales, unclassified Clostridiales, unclassified Erysipelotrichaceae, unclassified Lachnospiraceae, unclassified Mollicutes, unclassified Prevotellaceae and unclassified Rikenellaceae in RE was higher (*P* < 0.05) than those in faeces (Table 2), and the proportions of *Ruminococcus*,* Turicibacter*,* Clostridium*,* Cellulosilyticum*,* Akkermansia*, unclassified Peptostreptococcaceae and unclassified Ruminococcaceae were greater (*P* < 0.05) in faeces than in RE.

At the OTUs level, when compared with faeces (Table 3), the relative abundance of 17 OTUs was higher in RE (*P* < 0.05), while faeces showed a greater abundance of nine OTUs (*P* < 0.05).

### The density of the microbiota in RC, RE and faeces

The real‐time PCR results showed that the 16S rRNA gene copy numbers of total bacteria, phyla Firmicutes, and Bacteroidetes were higher (*P* < 0.01) in the RC and faeces than in RE (Table 4). The number of Firmicutes was greater (*P* < 0.01) in faeces than in RC, and no significant differences (*P* > 0.05) were observed in the numbers of total bacteria and Bacteroidetes between the RC and faeces.

**Table 4:** Population of total bacteria and phyla Bacteroidetes and Fimicutes in the RC, RE and faeces (*n* = 6) (log10 copy number of 16S RNA gene per gram of sample)

	RC	RE	Faeces	SEM^a^	*P*‐value
Total bacteria	12.05^a^	10.58^b^	12.33^a^	0.192	< 0.01
Firmicutes	11.08^a^	9.42^c^	11.60^b^	0.231	< 0.01
Bacteroides	11.84^a^	9.35^b^	11.46^a^	0.273	< 0.01

a Standard error of means.

Means without common letter differ, *P* < 0.05.

## Discussion

### Comparison of the composition of bacterial microbiota associated with the RC and RE

The RC‐associated microbial composition of dairy cows has been extensively surveyed using next‐generation sequencing technologies (Licht *et al*., 2007; Rettedal *et al*., 2009; Sayers *et al*., 2009; Callaway *et al*., 2010; Vasiljevic *et al*., 2011; Evans *et al*., 2012; Godoy‐Vitorino *et al*., 2012; Mao *et al*., 2013a; Mao *et al*., 2013b; Liu *et al*., 2014). In the present study, cows were slaughtered to collect enough of the epimural bacteria for a high‐quality community analysis. However, previous reports of bacterial community composition in slaughtered animals suffered from the fact that slaughter was performed long after the animals' last meal, which means that the RC bacterial community composition from slaughtered cows may be different from that from rumen fistulated cows. Current knowledge regarding the RE‐associated bacteria in dairy cattle is limited compared with the bacterial ecology and diversity of the RC microbial community. In this study, we hypothesized that the chosen sampling site can affect the diversity, composition and structure of bacteria microbiota in dairy cattle. In line with the reports based on PCR‐DGGE approach (Sadet *et al*., 2007), our results based on PCoA and AMOVA clearly demonstrated that the bacterial communities attached to RE were different from those attached to the RC (Fig. 1). Furthermore, quantification by qPCR gave higher values in terms of 16S rRNA genes in RC than RE for total bacteria. These distinctions between the bacterial community attached to the RE and those in RC may be explained based on sampling sites. The RE situated at the interface between the host tissues, while the RC is in contact with a variety of substrates and other microscale conditions, and this heterogeneous environment could affect bacterial diversity, composition and density as shown in other ecosystems (Horner‐Devine *et al*., 2004).

The differences in bacterial composition between RE and RC communities were studied at the phylum, genus and OTUs levels. At the phylum level, the abundance of Bacteroidetes was higher in the RC than in the RE of dairy cattle, and similar result was also observed in the 16S rRNA gene copy number between the RC and RE; this may be explained by the diversity of substrate utilization by Bacteroidetes spp. It is known that some Bacteroidetes species are able to hydrolyse soluble polysaccharides found in plant cell walls (Power *et al*., 2014). Thus, a higher abundance and number of Bacteroidetes spp. in RC will help to degrade and ferment organic matter (Salyers *et al*., 1995). Consistent with the reports by Chen *et al*. (2011), our study showed that RE contained a greater abundance of Proteobacteria, and this was believed to be caused by the trace amounts of oxygen diffused through the tissue, which may favour a high density of Proteobacteria as many members of this phylum are microaerophiles or facultative anaerobes and hence not sensitive to oxygen toxicity. Indeed, oxygen consumption by the epimural community is thought to be beneficial to the oxygen sensitive anaerobes of the rumen microbial ecosystem (Cheng *et al*., 1979).

At the genus level, *Prevotella* was predominant in the RC. Previous studies have revealed that different *Prevotella* spp. can utilize individual sugars, amino acids and small peptides for their growth (Fondevila and Dehority, 1996; Takahashi *et al*., 2000). Thus, they can selectively utilize carbohydrates and proteins from diet, which further resulted in predominance in rumen microbial community. In line with the report by Li *et al*. (2012), our results also revealed that the other abundant genera in the RC included *Succiniclasticum*,* Ruminococcus* and *Saccharofermentans*. These three genera are known to play an important role in fibre degradation (*Ruminococcus* and *Saccharofermentans*) and propionate formation (*Succiniclasticum*); thus, the fact that a higher percentage of these genera was observed in the RC is reasonable for dairy cattle. Conversely, the genera *Butyrivibrio*,* Campylobacter*,* Desulfobulbus*,* Syntrophococcus* and *Mogibacterium* were prevalent in RE. Of these, the abundance of *Butyrivibrio* (belonging to Firmicutes) was much higher in RE samples compared with the RC. These epithelial butyrate producers may release butyrate close to the epithelium and so they may enhance butyrate bioavailability for the host, which may be particularly useful in proliferating rumen and reticulum epithelium (Siavoshian *et al*., 2000). In addition, our results also revealed that the genus *Mogibacterium* dominated in RE, these results were consistent with the report by Li *et al*. (2012), who reported that the genus *Mogibacterium* was predominate in the RE of steers. This genus is associated with ammonia assimilation through the ruminal epithelial wall for phenylacetate biosynthesis (Nakazawa *et al*., 2000), and phenylacetate may bind to glutamine to form phenylacetyl glutamine by rumen bacteria, which is very important in rumen ammonia metabolism and absorption (Wallace, 1979). Our data also revealed that a higher proportion of *Campylobacter*, a microaerophilic genus, dominated of the RE compared to the RC in dairy cows, indicating that its presence is consistent with oxygen availability at the epithelial surface, as mentioned earlier. Consistent with a previous study by Mao *et al*. (2013b), the current study showed that some unclassified bacteria including unclassified Christensenellaceae, unclassified Rikenellaceae, unclassified Prevotellaceae, unclassified Ruminococcaceae and unclassified Bacteroidales were the predominant bacterial taxa in the RC and RE samples. In the present study, the reason that a higher abundance of unclassified Christensenellaceae, unclassified Ruminococcaceae and unclassified Bacteroidales was observed in RC samples remains unknown, but it is apparently because of their important role in the ruminal microbial ecosystem. As the meta‐analysis revealed that these unclassified bacterial groups were likely competitive in the rumen and that some of their species might have an important role in ruminal feed digestion (Kim *et al*., 2011). In summary, these data revealed that the composition and the abundance of dominate genera varied considerably between the RC and RE, and the unique distribution of epithelium‐attached bacterial composition is very likely due to host–bacterium interactions at the RE.

### Comparison of the composition of bacterial microbiota between the RC and faeces

The gastrointestinal tract serves as a habitat for a diverse and dynamic community of bacterial species that can affect growth, health and well‐being of the host. The faecal microbiota of cattle affects not only animal health but also food safety (Shanks *et al*., 2011). Recently, some research using pyrosequencing methods revealed that diet greatly influences the faecal microbiota of cattle (Callaway *et al*., 2010; Shanks *et al*., 2011). Although these studies provided some insights into the community structure of faecal microbiota, the number of sequences (< 10 000 reads) analysed in these studies was limited. Lack of power in deep sequencing prohibits an understanding of the whole profile of the faecal bacterial community. In the present study, a total of 265 156 sequences were obtained from the six samples (44 192 sequences per sample), and the coverage was higher than 0.97 (Table S2), which indicates that our study presented a relatively whole profile of the faecal microbial community.

In line with previous studies, Firmicutes in this study constituted a major fraction of the total sequencing reads in cattle faeces (Fig. 2A). At the genus level, our observations revealed a significantly higher prevalence of *Turicibacter* (belonging to Firmicutes), *Treponema* and *Clostridium* in the faecal microbial community compared with the RC. Of these three, *Turicibacter* is a relatively unstudied genus. Recent reports of 16S rRNA gene and ribosomal intergenic spacer analysis data indicate the presence of *Turicibacter* bacteria in the rumen and faeces of cattle (Callaway *et al*., 2010; Mao *et al*., 2013a). *Turicibacter* has also been reported in pig, rat and goat hindguts (Licht *et al*., 2007; Rettedal *et al*., 2009; Liu *et al*., 2014). Previous studies revealed that many *Treponema* spp. are associated with ulcerative mammary dermatitis and bovine digital dermatitis in cattle, and contagious ovine digital dermatitis in sheep (Sadet *et al*., 2007; Evans *et al*., 2012), which could have deleterious effects on the hindgut health. Conversely, some *Treponema* spp. can positively influence the host animal by improving the digestion of complex organic matter such as cellulose. Thus, the physiological function of this genus depends on which *Treponema* spp. are present. *Clostridium* spp. is a broad genus, and is ubiquitous in the gastrointestinal tract. Previous studies showed that Clostridia can both positively and negatively influence the host animal, and these effects are typically specifically associated with the individual *Clostridium* species that is involved (Kanauchi *et al*., 2005). Many have negative influences on animal health, including species such as *C. perfringens*,* C. tetani*,* C. botulinum* and *C. difficile* (Songer, 1998; Attwood *et al*., 2006), and they can also cause significant productivity problems including reducing the protein availability in fresh forage diets (Reilly and Attwood, 1998). Conversely, some *Clostridium* spp. may be beneficial and improve the digestion of complex organic matter such as cellulose, or even act as beneficial probiotics (Widyastuti *et al*., 1992; Ozutsumi *et al*., 2005).

Our results also revealed that the dominant genera *Prevotella*,* Succiniclasticum*,* Ruminococcus*,* Acetitomaculum*,* Saccharofermentans*,* Mogibacterium* and some unclassified bacteria including unclassified Christensenellaceae, unclassified Bacteroidales, unclassified Rikenellaceae, unclassified Lachnospiraceae, unclassified Prevotellaceae and unclassified Mollicutes were much higher in RC than in faeces, while the *Turicibacter*,* Clostridium* and unclassified Peptostreptococcaceae‐dominated faeces, suggesting that the predominant community of microbial flora were different between the RC and faeces. In addition, a PCoA and AMOVA revealed that the structure of the ruminal bacterial community was significantly different from the faecal microbiota. However, despite these differences in the bacterial community of the microhabitats, no significant difference was found in the level of total volatile fatty acids between the RC and faeces (Fig. S4), indicating that faecal microbial communities tend to exhibit a similar function during fermentation of some substrates.

In the present study, our data showed that 1260 OTUs were shared between the rumen microbiota and the faecal microbiota (Fig. S4), indicating that these OTUs detected in the rumen might survive in the hindgut. Nevertheless, it is worthy of note that this speculation is based on a PCR assay, and an important drawback to PCR is the potential amplification of DNA from dead bacterial cells as well as from viable bacterial cells (Josephson *et al*., 1993). Thus, part of the core OTUs detected in the hindgut might be from dead cells. In addition, as most of the shared OTUs cannot be identified at the genus level, the function of these species is not very clear. Therefore, new methods such as proteomics and transcriptomics methods should be applied to elucidate their function and activity in the cattle gastrointestinal microbial ecosystem.

In conclusion, our findings clearly demonstrated the striking compositional differences between RC, RE and faeces, indicating that bacterial communities are specific and adapted to the harbouring environment.

## Funding information

No funding information provided.

## Conflicts of interest

None of the authors of this paper has a financial or personal relationship with other people or organizations that could inappropriately influence or bias the content of the paper.

## Supporting information


**Table S1.** Ingredients and nutrients of the experimental diets.
**Table S2.** Number of sequences, estimated sample coverage, diversity and OTU richness at 3 % dissimilarity level in each sample
**Fig. S1.** Rarefaction curves of OTUs defined by 3%, 5% and 10% distances for each samples. Rumen contents (RC): RC‐1, RC‐2, RC‐3, RC‐4, RC‐5, RC‐6; Rumen epithelium (RE): RE‐1, RE‐2, RE‐3, RE‐4, RE‐5, RE‐6; Feces: Feces‐1, Feces‐2, Feces‐3, Feces‐4, Feces‐5, Feces‐6.
**Fig. S2.** Influence of sampling sites on microbiota of rumen content (RC), rumen epithelium (RE) and feces of dairy cattle for the top 50 most abundant genera. RC: RC‐1, RC‐2, RC‐3, RC‐4, RC‐5, RC‐6; RE: RE‐1, RE‐2, RE‐3, RE‐4, RE‐5, RE‐6; Feces: Feces‐1, Feces‐2, Feces‐3, Feces‐4, Feces‐5, Feces‐6.
**Fig. S3.** Venn diagram of the overlap between observed OTUs at 3% divergence in rumen content (RC), rumen epithelium (RE), feces. Data are also represented by the phyla to which the detected unique OTUs belong.
**Fig. S4.** The concentrations of volatile fatty acids in the rumen content (RC) and feces.
