# Supplementary material for: Comparative studies of the composition of bacterial microbiota associated with the ruminal content, ruminal epithelium and in the faeces of lactating dairy cows
Source: Microb Biotechnol. 2016 Feb 1;9(2):257–68. doi: 10.1111/1751-7915.12345 (PMC4767291; doi:10.1111/1751-7915.12345)
Supplement: Supplementary file 1 — Table S1. Ingredients and nutrients of the experimental diets. Table S2. Number of sequences, estimated sample coverage, diversity and OTU richness at 3 % dissimilarity level in each sample Fig. S1. Rarefaction curves of OTUs defined by 3%, 5% and 10% distances for each samples. Rumen contents (RC): RC‐1, RC‐2, RC‐3, RC‐4, RC‐5, RC‐6; Rumen epithelium (RE): RE‐1, RE‐2, RE‐3, RE‐4, RE‐5, RE‐6; Feces: Feces‐1, Feces‐2, Feces‐3, Feces‐4, Feces‐5, Feces‐6. Fig. S2. Influence of sampling sites on microbiota of rumen content (RC), rumen epithelium (RE) and feces of dairy cattle for the top 50 most abundant genera. RC: RC‐1, RC‐2, RC‐3, RC‐4, RC‐5, RC‐6; RE: RE‐1, RE‐2, RE‐3, RE‐4, RE‐5, RE‐6; Feces: Feces‐1, Feces‐2, Feces‐3, Feces‐4, Feces‐5, Feces‐6. Fig. S3. Venn diagram of the overlap between observed OTUs at 3% divergence in rumen content (RC), rumen epithelium (RE), feces. Data are also represented by the phyla to which the detected unique OTUs belong. Fig. S4. The concentrations of volatile fatty acids in the rumen content (RC) and feces. [file MBT2-9-257-s001.docx]

**Description**

**Table S1.** Ingredients and nutrients of the experimental diets.

**Table S2.** Number of sequences, estimated sample coverage, diversity and OTU richness at 3 % dissimilarity level in each sample.

**Fig. S1.** Rarefaction curves of OTUs defined by 3%, 5% and 10% distances for each samples. Rumen contents (RC): RC-1, RC-2, RC-3, RC-4, RC-5, RC-6; Rumen epithelium (RE): RE-1, RE-2, RE-3, RE-4, RE-5, RE-6; Feces: Feces-1, Feces-2, Feces-3, Feces-4, Feces-5, Feces-6.

**Fig. S2.** Influence of sampling sites on microbiota of rumen content (RC), rumen epithelium (RE) and feces of dairy cattle for the top 50 most abundant genera. RC: RC-1, RC-2, RC-3, RC-4, RC-5, RC-6; RE: RE-1, RE-2, RE-3, RE-4, RE-5, RE-6; Feces: Feces-1, Feces-2, Feces-3, Feces-4, Feces-5, Feces-6.

**Fig. S3.** Venn diagram of the overlap between observed OTUs at 3% divergence in rumen content (RC), rumen epithelium (RE), feces. Data are also represented by the phyla to which the detected unique OTUs belong.

**Fig. S4.** The concentrations of volatile fatty acids in the rumen content (RC) and feces.

**Table S1.** Ingredients and nutrients of the experimental diets

| Ingredients | Composition (%) | Nutrients | Contents |
| --- | --- | --- | --- |
| Ground corn grain | 27.0 | Dry matter (DM), % | 54.3 |
| Wheat bran | 5.1 | Organic matter, % of DM | 91.7 |
| Soybean meal | 12.7 | Crude protein, % of DM | 16.2 |
| Cottonseed meal | 4.3 | Neutral detergent fiber, % of DM | 36.3 |
| Corn silage | 15 | Acid detergent fiber, % of DM | 19.5 |
| Corn stover | 30 | Non-fiber carbohydrate, % of DM | 36.0 |
| Urea | 1 | Calcium, % of DM | 0.62 |
| Premix^1^ | 4.9 | Phosphorus, % of DM | 0.45 |
|  |  | Lysine, % of DM | 0.69 |
|  |  | Methionine, % of DM | 0.13 |
|  |  | Net energy lactation , Mcal/kg | 1.45 |

^1^Formulated to provide (per kilogram of DM): 174 g of zeolite powder, 1.25 g of yeast, 25 g of mold adsorbent (Solis Mos, Novus International Inc., St. Charles, MO), 21.44 g of KCl, 41.25 g of MgO, 150 g of Salt, 187.5 g of NaHCO3, 84 g of Ca, 15 g of P, 125,000 IU of vitamin A, 750,000 IU of vitamin D3, 937.5 IU of vitamin E, 1750 mg of Zn, 17.5 mg of Se, 28.75 mg of I, 375 mg of Fe, 15 mg of Co, 556.5 mg of Mn and 343.75 mg of Cu

**Table S2.** Number of sequences, estimated sample coverage, diversity and OTU richness at 3 % dissimilarity level in each sample.

| Sample ID | Valid sequences | The number of OTUs | Chao 1 value | Shannon index | Coverage |
| --- | --- | --- | --- | --- | --- |
| Rumen content-1 | 36231 | 1970 | 2428 | 6.13 | 0.99 |
| Rumen content-2 | 38802 | 2007 | 2463 | 6.14 | 0.99 |
| Rumen content-3 | 52094 | 2116 | 2481 | 6.06 | 0.99 |
| Rumen content-4 | 35196 | 2042 | 2711 | 5.96 | 0.98 |
| Rumen content-5 | 38143 | 1909 | 2319 | 6.01 | 0.99 |
| Rumen content-6 | 35488 | 1949 | 2458 | 6.1 | 0.99 |
| Feces-1 | 55255 | 1353 | 1768 | 4.17 | 0.99 |
| Feces-2 | 41317 | 1274 | 1639 | 4.29 | 0.99 |
| Feces-3 | 40864 | 1223 | 1953 | 3.83 | 0.99 |
| Feces-4 | 46474 | 1368 | 1725 | 4.62 | 0.99 |
| Feces-5 | 40750 | 1184 | 1600 | 4.19 | 0.99 |
| Feces-6 | 40496 | 1324 | 1710 | 4.72 | 0.99 |
| Rumen epithelium-1 | 63434 | 2152 | 2721 | 5.42 | 0.99 |
| Rumen epithelium-2 | 49493 | 1892 | 2393 | 5.7 | 0.99 |
| Rumen epithelium-3 | 58340 | 1668 | 2066 | 5.34 | 0.99 |
| Rumen epithelium-4 | 61390 | 2111 | 2623 | 5.54 | 0.99 |
| Rumen epithelium-5 | 51927 | 1948 | 2481 | 5.43 | 0.99 |
| Rumen epithelium-6 | 50052 | 1896 | 2350 | 5.56 | 0.99 |

**Fig. S1**. Rarefaction curves of OTUs defined by 3%, 5% and 10% distances for each samples. Rumen contents (RC): RC-1, RC-2, RC-3, RC-4, RC-5, RC-6; Rumen epithelium (RE): RE-1, RE-2, RE-3, RE-4, RE-5, RE-6; Feces: Feces-1, Feces-2, Feces-3, Feces-4, Feces-5, Feces-6

**
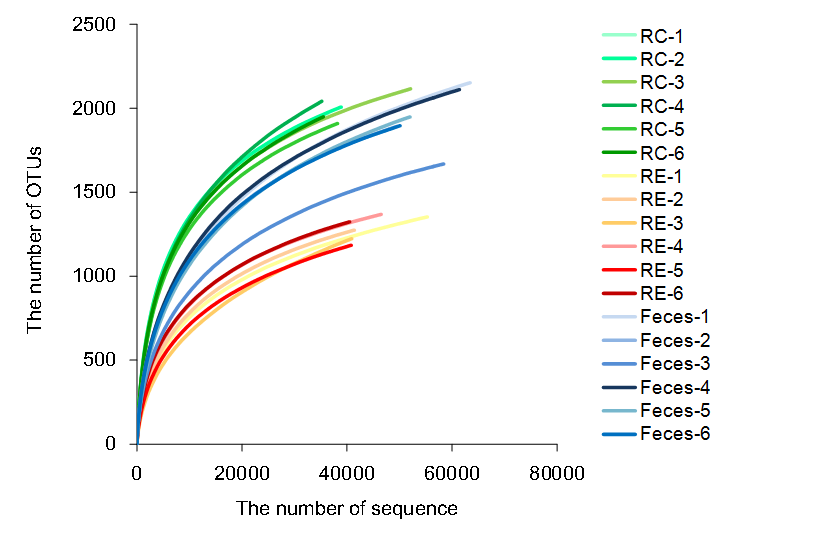
**

**Fig. S2**. Influence of sampling sites on microbiota of rumen content (RC), rumen epithelium (RE) and feces of dairy cattle for the top 50 most abundant genera. RC: RC-1, RC-2, RC-3, RC-4, RC-5, RC-6; RE: RE-1, RE-2, RE-3, RE-4, RE-5, RE-6; Feces: Feces-1, Feces-2, Feces-3, Feces-4, Feces-5, Feces-6

**Fig. S3.** Venn diagram of the overlap between observed OTUs at 3% divergence in rumen content (RC), rumen epithelium (RE), feces. Data are also represented by the phyla to which the detected unique OTUs belong.

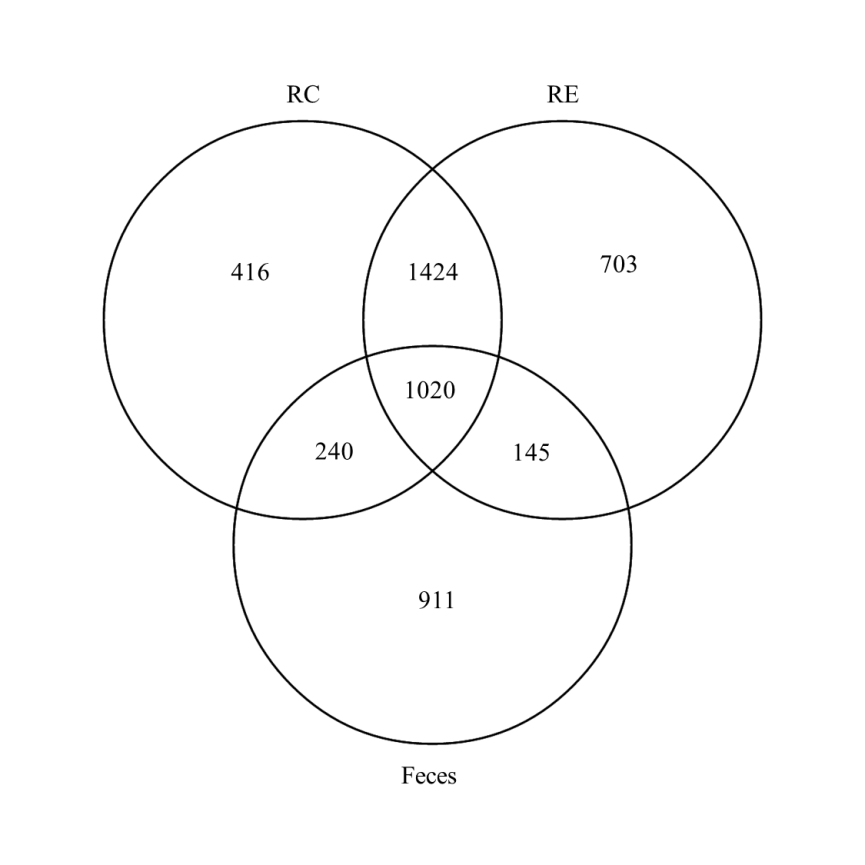

**Fig. S4.** The concentrations of volatile fatty acids in the rumen content (RC) and feces.
